# Patterns of multimorbidity and risk of disability in community-dwelling older persons

**DOI:** 10.1007/s40520-020-01773-z

**Published:** 2021-02-13

**Authors:** Alessandra Marengoni, Roselyne Akugizibwe, Davide L. Vetrano, Albert Roso-Llorach, Graziano Onder, Anna-Karin Welmer, Amaia Calderón-Larrañaga

**Affiliations:** 1 Department of Clinical and Experimental Sciences, University of Brescia, Brescia, Italy; 2 Aging Research Center, Department of Neurobiology, Care Sciences and Society, Karolinska Institutet and Stockholm University, Stockholm, Sweden; 3 Centro Medicina Dell’Invecchiamento, Fondazione Policlinico Universitario “A. Gemelli” IRCCS, Università Cattolica del Sacro Cuore, Rome, Italy; 4 Fundació Institut Universitari per a la Recerca a l’Atenció Primària de Salut Jordi Gol i Gurina (IDIAPJGol), Gran Via Corts Catalanes 587, Barcelona, Spain; 5 Universitat Autònoma de Barcelona, Campus de la UAB, Bellaterra (Cerdanyola del Vallès), Spain; 6 Department of Cardiovascular, Endocrine-Metabolic Diseases and Aging, Istituto Superiore Di Sanità, Rome, Italy; 7 Division of Physiotherapy, Department of Neurobiology, Care Sciences and Society, Karolinska Institutet, Stockholm, Sweden; 8 Karolinska University Hospital, Stockholm, Sweden; 9 Stockholm Gerontology Research Center, Stockholm, Sweden

**Keywords:** Multimorbidity patterns, Disability, Older persons

## Abstract

The aim was to analyze the association between specific patterns of multimorbidity and risk of disability in older persons. Data were gathered from the Swedish National Study on Aging and Care in Kungsholmen (SNAC-K); 2066 60 + year-old participants living in the community and free from disability at baseline were grouped according to their multimorbidity patterns and followed-up for six years. The association between multimorbidity patterns and disability in basic (ADL) and instrumental (IADL) activities of daily living was examined through multinomial models. Throughout the follow-up, 434 (21.0%) participants developed at least one ADL and 310 (15.0%) at least one IADL. Compared to the *unspecific* pattern, which included diseases not exceeding their expected prevalence in the total sample, belonging to the *cardiovascular/anemia/dementia,* the *sensory impairment/cancer* and the *musculoskeletal/respiratory/gastrointestinal* patterns was associated with a higher risk of developing both ADL and IADL, whereas subjects in the *metabolic/sleep disorders* pattern showed a higher risk of developing only IADL. Multimorbidity patterns are differentially associated with incident disability, which is important for the design of future prevention strategies aimed at delaying functional impairment in old age, and for a better healthcare resource planning.

**Supplementary information:**

The online version contains supplementary material available at (10.1007/s40520-020-01773-z).

## Introduction

The co-occurrence of multiple diseases in the same individual, i.e., multimorbidity, is the most prevalent and burdensome condition in older adults [1], accounting for many years of life lived with disability [2] and functional decline over time [3, 4]. Despite its common use in research, assessing the impact of simple counts of chronic diseases on health outcomes may be insufficient to explain the heterogeneity across older persons’ functional status. In the past few years, patterns of systematically clustering diseases have been increasingly investigated, in an attempt to explain their potential synergistic effects on several health outcomes [5, 6]. In previous reports from our group, we showed that persons affected by specific multimorbidity patterns have increased risk of death [7] and dementia onset [6], compared to those belonging to an unspecific pattern.

Similarly, persons affected by specific patterns of multimorbidity may be expected to differ in their risk of developing functional decline and disability over time, but only few studies have evaluated the association between multimorbidity patterns and disability, mostly at a cross-sectional level [5]. In the Health and Retirement Study, subjects within a pattern comprising arthritis, hypertension and depression showed the highest level of limitations in basic and instrumental activities of daily living compared to healthy participants [8]. In another study, the combination of somatic and mental diseases was associated with substantially greater prospective disability than combinations comprised exclusively of somatic conditions [9].

The aim of this study was to analyze the association between specific patterns of multimorbidity and the six-year risk of disability in community-dwelling Swedish older persons.

## Methods

### Study population

We carried out a prospective cohort study using data from the Swedish National study on Aging and Care in Kungsholmen (SNAC-K). SNAC-K is an ongoing longitudinal study including people aged 60 + years (range: 60–104) living either in the community or in institutions in the Kungsholmen central area of Stockholm, Sweden [10]. Participants were randomly selected from 11 age cohorts (60, 66, 72, 78, 81, 84, 87, 90, 93, 96 and 99 + years) during the baseline wave (2001–2004), leading to a study population of 3363 individuals (response rate: 73.3%). Follow-up interviews are performed every 3 years for individual 78 years and above and every 6 years for individuals younger than 78 years.

The present study included data from 2741 individuals with multimorbidity (i.e., 2 + chronic diseases) and living in the community at baseline, who were followed-up for 6 years. We excluded: (1) individuals with any limitations in basic or instrumental activities of daily living (i.e., ADL and IADL) at baseline (*n* = 592), (2) subjects receiving formal home care at baseline (*n* = 51), and (3) participants with no data for ADL or IADL at follow-up (*n* = 32). The analytical sample consisted of 2066 participants.

Ethical approval was obtained from the Regional Ethics Review Board in Stockholm County, and informed consent was obtained from participants or a proxy (if the participant had severe cognitive impairment) before participation in the study.

### Chronic disease assessment

This was done using participants’ medical history, clinical examination, inpatient and outpatient registers, self-reported information and proxy interviews. In addition, laboratory tests and medications were used for the diagnosis of specific conditions. Chronic diseases were coded according to the International Classification of Diseases, 10th revision (ICD10) and were grouped into 60 categories based on an operationalization that has been developed and applied to SNAC-K data in a previous proof-of-concept study (1). The clinical criteria for grouping the identified diagnoses included similarities in their pathophysiology, treatment, prognosis and prevalence.

### Disability assessment

The primary outcomes were incident disability in at least one ADL (i.e., bathing, dressing, toilet use, urinary continence, transferring self from bed and eating) and IADL (i.e., phone use, grocery shopping, food preparation, housekeeping, laundry, use of public transport, handling finances and medication use). These were assessed using a questionnaire administered by a qualified nurse, and each item was graded as “independent” or “dependent.”

### Covariates

Data on age, sex, education, marital status, living arrangement, informal and formal care were gathered through questionnaires. Education was measured as the highest attained level. Formal social care includes help with household chores and personal care. It is financed by the municipality and may be delivered either by the municipality or a private company that has a contract with the municipality. Informal social care covers service or care assistance from relatives, friends, neighbors, or volunteer/non-profit organizations. The amount of formal and informal care a person received was recorded as hours per month.

### Analysis

We employed a fuzzy c-means cluster analysis to classify the study participants into six homogeneous patterns of multimorbidity: *psychiatric diseases; cardiovascular diseases, anemia and dementia; metabolic and sleep disorders; sensory impairments and cancer; musculoskeletal, respiratory and gastrointestinal diseases;* and *unspecific* (Table S1). Participants in the *unspecific* pattern had diseases that did not specifically characterize this pattern [11].

We used multinomial logistic regression models to test the association between multimorbidity patterns and the different outcomes, and results were reported as relative rate ratios (RRR) with their confidence intervals. Secondary outcomes were: (1) death during follow-up, which was derived from the Swedish death register, and (2) loss to follow-up. Potential interactions between multimorbidity patterns and sex were assessed, and sex-stratified analyses were carried out subsequently. Analyses were carried out using Stata IC/15.0, and a *P*-value of < 0.05 was considered statistically significant.

## Results

This study consisted of 1267 (61.3%) participants aged < 78 years and 799 (38.7%) participants aged ≥ 78 years (Table 1). Most of the participants in the study were female (*n* = 1298, 62.8%). Participants were distributed across the six different multimorbidity patterns as follows: *psychiatric* (5.3%), *cardiovascular/anemia/dementia* (4.6%), *metabolic/sleep disorders* (12.5%), *sensory impairment/cancer* (8.6%), *musculoskeletal/respiratory/gastrointestinal* (15.5%) and an *unspecific* pattern (53.4%) including diseases not exceeding their expected prevalence in the total sample. By the end of the 6-year follow-up, 434 (21.0%) of the participants developed at least one ADL. Within this group of subjects, 58.5% were < 78 years, the majority were female (80.6%) and most had ≥ 4 chronic conditions (58.5%). During the same period, 310 (15.0%) participants developed at least one IADL; they were mostly aged ≥ 78 years (79.3%) and females (70.3%), 51.84% had polypharmacy and most had ≥ 4 chronic conditions (73.6%).

**Table 1:** Baseline characteristics of participants by the different outcomes

Characteristics	Total *N* = 2066	ADL disability *n* = 434	No ADL disability *n* = 1,027	*P*-value	IADL disability *n* = 310	No IADL disability *n* = 1,205	*P*-value
Age, *n* (%)				< 0.001			< 0.001
< 78 years	1,267 (61.3)	254 (58.5)	747 (72.7)		64 (20.6)	937 (77.8)	
≥ 78 years	799 (38.7)	180 (41.5)	280 (27.3)		246 (79.4)	268 (22.2)	
Gender, *n* (%)				< 0.001			< 0.003
Males	768 (37.2)	84 (19.4)	457 (44.5)		92 (29.7)	464 (38.5)	
Females	1,298 (62.8)	350 (80.6)	570 (55.5)		218 (70.3)	741 (61.5)	
Education, *n* (%)				< 0.001			< 0.001
Elementary	294 (14.3)	63 (14.5)	112 (10.9)		68 (22.1)	122 (10.1)	
High school	1,036 (50.3)	223 (51.5)	491 (47.9)		166 (53.9)	580 (48.1)	
University	728 (35.4)	147 (33.9)	423 (41.2)		74 (24.0)	503 (41.7)	
Marital status, *n* (%)				< 0.001			< 0.001
Unmarried	340 (16.5)	72 (16.6)	173 (16.9)		40 (12.9)	210 (17.4)	
Married	970 (47.2)	188 (43.3)	544 (53.0)		114 (36.8)	635 (52.7)	
Divorced	302 (14.7)	70 (16.1)	149 (14.5)		41 (13.2)	182 (15.1)	
Widow	445 (21.6)	104 (24.0)	160 (15.6)		115 (37.1)	177 (14.7)	
Polypharmacy, *n* (%)				< 0.001			< 0.001
≥ 5 drugs	701 (33.9)	185 (42.6)	269 (26.2)		157 (50.6)	324 (26.9)	
≥ 10 drugs	103 (5.0)	27 (6.2)	31 (3.0)		31 (10.0)	33 (2.7)	
Chronic diseases, *n* (%)				< 0.001			< 0.001
≥ 3 chronic diseases	1,558 (75.4)	355 (81.8)	713 (69.4)		276 (89.0)	845 (70.1)	
≥ 4 chronic diseases	1,046 (50.6)	254 (58.5)	452 (44.0)		228 (73.5)	524 (43.5)	
Multimorbidity patterns, *n* (%)				< 0.001			< 0.001
Psychiatric	109 (5.3)	27 (6.2)	51 (5.0)		15 (4.8)	65 (5.4)	
Cardio/Anemia/Dementia	96 (4.6)	18 (4.1)	24 (2.3)		26 (8.4)	24 (2.0)	
Metabolic/Sleep	259 (12.5)	47 (10.8)	116 (11.3)		37 (11.9)	135 (11.2)	
Sensory/Cancer	178 (8.6)	47 (10.8)	71 (6.9)		56 (18.1)	72 (6.0)	
MSK/Resp/GI	320 (15.5)	83 (19.1)	143 (13.9)		65 (21.0)	170 (14.1)	
Unspecific	1,104 (53.4)	212 (48.8)	622 (60.6)		111 (35.8)	739 (61.3)	

Test: Pearson's chi-squared

Missing data: education 8, marital status 9, informal care 33

Abbreviations: *GI* gastro-intestinal diseases, *Resp* respiratory diseases, *MSK* musculoskeletal, *Cardio* cardiovascular diseases

Belonging to the *cardiovascular/anemia/dementia* (RRR 2.09, 95% CI 1.08, 4.06)*,* the *sensory impairment/cancer* (RRR 1.62, 95% CI 1.05, 2.48) and the *musculoskeletal/respiratory/gastrointestinal* (RRR 1.40, 95% CI 1.02, 1.94) patterns was significantly associated with a higher risk of developing ADL, compared to the *unspecific* pattern (Table 2). Moreover, subjects in the *cardiovascular/anemia/dementia* pattern (RRR 3.11, 95% CI 1.64, 5.89), the *sensory impairment/cancer* pattern (RRR 2.11, 95% CI 1.36, 3.28), the *musculoskeletal/respiratory/gastrointestinal* pattern (RRR 1.97, 95% CI 1.34, 2.90), and those in the *metabolic/sleep disorders* pattern (RRR 1.58, 95% CI 1.00, 2.48) showed a higher risk of developing IADL compared with those in the *unspecific* pattern (Table 2).

**Table 2:** Association (RRR and 95%CI) of multimorbidity patterns with the risk of developing ADL and IADL

Multimorbidity patterns	Cases/at risk	RRR (95% CI)
*ADL*		
Unspecific	212/1104	Ref.
Psychiatric	27/109	1.50 (0.91, 2.49)
Cardio/Anemia/Dementia	18/96	2.09 (1.08, 4.06)*
Metabolic/Sleep	47/259	1.41 (0.95, 2.07)
Sensory/Cancer	47/178	1.62 (1.05, 2.48)*
MSK/Resp/GI	83/320	1.40 (1.02, 1.94)*
*IADL*		
Unspecific	111/1104	Ref.
Psychiatric	15/109	1.79 (0.93, 3.43)
Cardio/Anemia/Dementia	26/96	3.11 (1.64, 5.89)***
Metabolic/Sleep	37/259	1.58 (1.00, 2.48)*
Sensory/Cancer	56/178	2.11 (1.36, 3.28)***
MSK/Resp/GI	65/320	1.97 (1.34, 2.90)***

Model adjusted by age, sex, education level, marital status

Abbreviations: *RRR* relative rate ratio, *GI* gastro-intestinal diseases, *Resp* respiratory diseases, *MSK* musculoskeletal, *Cardio* cardiovascular diseases, *CI* confidence interval

*P*-values: *< 0.05, **≤ 0.01, ***≤ 0.001

There was no statistically significant interaction between the multimorbidity patterns and sex. Sex stratification strengthened the association between the *cardiovascular/anemia/dementia* pattern and the risk of developing ADL among females (RRR 3.42, 95% CI 1.42, 8.22), while the association with other patterns was weakened and non-significant. Among males, the risk of developing ADL was strengthened for the *sensory impairment/cancer* pattern (RRR 2.42, 95% CI 1.14, 5.15), but no significant associations were observed with the other patterns. For IADL, with the exception of the *metabolic/sleep disorder* pattern, the associations were strengthened for all the patterns among females, including the *psychiatric* pattern, which became statistically significant (RRR 2.20, 95% CI 1.02, 4.73). On the contrary, the associations with all patterns were attenuated and became non-significant among males (Table S2).

The association between the multimorbidity patterns and death or dropout can be found as supplementary material (Table S3).

## Discussion

Compared to the *unspecific* pattern, belonging to the *cardiovascular/anemia/dementia* pattern, the *sensory impairment/cancer* pattern and the *musculoskeletal/respiratory/gastrointestinal* pattern was significantly associated with a higher risk of developing both ADL and IADL, whereas subjects in the *metabolic/sleep disorders* pattern showed a higher risk of developing only IADL. Furthermore, in stratified analyses, the association between the *cardiovascular/anemia/dementia* pattern and the risk of developing ADL was strengthened in women, and that between the *sensory impairment/cancer* pattern and ADL was strengthened in men, whereas the associations with IADL development were only significant in women. These findings confirm the complexity and heterogeneity of multimorbidity and its consequences for older persons’ health status, suggesting the need to go beyond the simple count of chronic conditions and its link with increased risk of functional decline, as previously established [3].

Studies evaluating the topic are few and used different methodologies in analyzing disease clustering. In the Australian Longitudinal Study on Women’s Health, where multimorbidity patterns were identified using factor analysis, the authors found that the cardiovascular pattern was associated with the greatest decline in ADL over six years, whereas the neurological/mental health pattern was associated with the greatest decline in IADL, compared to a group of women that did not have multimorbidity at baseline or that were multimorbid but their conditions did not group into any of the identified patterns [12]. Similarly, we compared specific multimorbidity patterns with an unspecific one including diseases that did not exceed the expected prevalence in the total sample.

Several hypotheses can be suggested concerning the differential risk of disability associated with multimorbidity patterns. First, the development of chronic diseases could be an intermediate link among various pathophysiological processes happening in older age. In fact, aging-related biological processes such as chronic inflammation and cellular senescence may represent the root mechanisms leading to disability through chronic diseases [13]. In a previous report, we showed that inflammatory marker levels differ according to specific patterns of multimorbidity, being highest in the cardiovascular multimorbidity pattern [11]. Another hypothesis is that the combinations of specific diseases may enhance sarcopenia and the associated disability onset [14], which could be especially relevant in the case of the pattern including musculoskeletal and respiratory diseases, as both conditions are often characterized by loss of lean muscle mass [15, 16]. One additional possible theory is that combinations of diseases that affect functional ability through different mechanisms may have a worse effect on functional decline than comorbidities sharing common etiologic or pathophysiologic mechanisms [17]. In fact, our findings showed that the pattern including conditions affecting three different organ systems, i.e., cardiac diseases, dementia and anemia, was one of the most burdensome in terms of associated disability risk. Finally, recent evidence suggests that psychiatric disturbances, such as generalized anxiety disorders, are more strongly associated with disability in women than in men [18]. This is in line with our finding whereby the psychiatric pattern was only associated with incident disability in females. 

Among the limitations of this study are the fact that the main exposure (i.e., multimorbidity patterns) was only measured at baseline, and the limited transportability of the findings, given that SNAC-K participants are on average wealthier and healthier than the general older population in Sweden. Our strict exclusion criteria is likely to have limited the generalizability of our findings.

## Conclusions

Findings from our study suggest that specific multimorbidity patterns may differentially lead to functional decline, which is important for better understanding the relationship between diseases and disability, for the design of future prevention strategies aimed at delaying functional impairment in old age, and for a better healthcare resource planning.

## Supplementary information

Below is the link to the electronic supplementary material.

Supplementary file1 (DOCX 30 kb)
