# Supplementary material for: Patterns of multimorbidity and risk of disability in community-dwelling older persons
Source: Aging Clin Exp Res. 2021 Feb 13;33(2):457–62. doi: 10.1007/s40520-020-01773-z (PMC7914228; doi:10.1007/s40520-020-01773-z)
Supplement: Supplementary file 1 — Supplementary file1 (DOCX 30 kb) [file 40520_2020_1773_MOESM1_ESM.docx]

**Table S1. Multimorbidity patterns identified through fuzzy c-mean cluster analysis.**

| **Multimorbidity**  **pattern** | **Diseases** | **Prevalence within pattern (%)** | **Observed / Expected Ratio** | **Exclusivity (%)** |
| --- | --- | --- | --- | --- |
| **Psychiatric** | | | | |
|  | Neurotic, stress-related and somatoform diseases | 54.45 | 15.55 | 94.52 |
|  | Depression and mood diseases | 82.57 | 8.20 | 49.86 |
|  | Other neurological diseases | 6.00 | 2.89 | 17.55 |
|  | Asthma | 17.54 | 2.45 | 14.91 |
|  | Sleep disorders | 5.48 | 2.28 | 13.84 |
|  | Colitis and related diseases | 26.82 | 2.23 | 13.59 |
|  | Migraine and facial pain syndromes | 4.95 | 1.97 | 11.95 |
|  | Other musculoskeletal and joint diseases | 12.50 | 1.80 | 10.97 |
|  | COPD, emphysema, chronic bronchitis | 9.12 | 1.65 | 10.06 |
|  | Oesophagus, stomach and duodenum diseases | 7.86 | 1.58 | 9.63 |
| **Cardiovascular, Anemia & Dementia** | | | | |
|  | Bradycardias and conduction diseases | 18.88 | 9.08 | 79.84 |
|  | Other cardiovascular diseases | 25.51 | 6.92 | 60.87 |
|  | Heart failure | 71.59 | 6.52 | 57.32 |
|  | Cardiac valve diseases | 17.82 | 6.11 | 53.68 |
|  | Atrial fibrillation | 58.23 | 5.54 | 48.73 |
|  | Cerebrovascular disease | 26.74 | 3.39 | 29.84 |
|  | Ischemic heart disease | 51.85 | 3.07 | 26.99 |
|  | Anemia | 37.25 | 2.84 | 25.00 |
|  | Dementia | 17.20 | 2.84 | 24.97 |
|  | Inflammatory arthropathies | 11.71 | 2.57 | 22.57 |
| **Sensory & Cancer** | | | | |
|  | Cataract and other lens diseases | 35.98 | 5.87 | 68.86 |
|  | Blindness, visual impairment | 24.40 | 5.72 | 67.06 |
|  | Other eye diseases | 33.09 | 5.70 | 66.92 |
|  | Glaucoma | 26.79 | 4.42 | 51.89 |
|  | Deafness, hearing impairment | 34.82 | 3.00 | 35.21 |
|  | Solid neoplasms | 21.93 | 2.15 | 25.19 |
|  | Other genitourinary diseases | 5.67 | 1.97 | 23.08 |
|  | Dementia | 11.83 | 1.95 | 22.91 |
|  | Anemia | 22.77 | 1.74 | 20.39 |
|  | Chronic kidney diseases | 65.36 | 1.69 | 19.85 |
| **Metabolic & Sleep** | | | | |
|  | Diabetes | 37.96 | 3.77 | 42.31 |
|  | Sleep disorders | 7.62 | 3.16 | 35.50 |
|  | Obesity | 38.64 | 2.79 | 31.28 |
|  | Ischemic heart disease | 33.50 | 1.98 | 22.26 |
|  | Inflammatory arthropathies | 9.02 | 1.98 | 22.20 |
|  | Other cardiovascular diseases | 6.34 | 1.72 | 19.31 |
|  | Dementia | 10.29 | 1.70 | 19.08 |
|  | Prostate diseases | 7.56 | 1.63 | 18.30 |
|  | COPD, emphysema, chronic bronchitis | 8.17 | 1.48 | 16.65 |
|  | Heart failure | 16.13 | 1.47 | 16.49 |
| **Musculoskeletal, Respiratory & Gastro-intestinal** | | | | |
|  | Osteoporosis | 31.41 | 4.26 | 67.29 |
|  | Dorsopathies | 21.80 | 2.93 | 46.23 |
|  | Oesophagus, stomach and duodenum diseases | 14.21 | 2.86 | 45.20 |
|  | Asthma | 19.98 | 2.79 | 44.10 |
|  | COPD, emphysema, chronic bronchitis | 12.77 | 2.32 | 36.59 |
|  | Autoimmune diseases | 11.67 | 2.28 | 36.07 |
|  | Inflammatory arthropathies | 9.07 | 1.99 | 31.39 |
|  | Thyroid diseases | 22.74 | 1.96 | 30.95 |
|  | Colitis and related diseases | 22.95 | 1.91 | 30.19 |
|  | Osteoarthritis and other degenerative joint diseases | 27.58 | 1.88 | 29.61 |
| **Unspecific** | | | | |
|  | Dyslipidemia | 67.35 | 1.28 | 59.30 |
|  | Hypertension | 84.61 | 1.12 | 51.82 |
|  | Obesity | 13.07 | 0.94 | 43.71 |
|  | Prostate diseases | 4.29 | 0.93 | 42.92 |
|  | Other musculoskeletal and joint diseases | 5.83 | 0.84 | 39.00 |
|  | Other genitourinary diseases | 2.42 | 0.84 | 38.96 |
|  | Solid neoplasms | 7.86 | 0.77 | 35.69 |
|  | Migraine and facial pain syndromes | 1.88 | 0.75 | 34.64 |
|  | Thyroid diseases | 8.56 | 0.74 | 34.23 |
|  | Osteoarthritis and other degenerative joint diseases | 10.63 | 0.72 | 33.53 |

Abbreviation: COPD – Chronic Obstructive Pulmonary Disease

Diseases were included in specific patterns if their observed/expected ratio was ≥2 OR exclusivity ≥25%.

Diseases with both observed/expected ratio ≥2 AND exclusivity ≥25% were considered to characterize each cluster (grey bands).

**Table S2. Association (RRR and 95%CI) of multimorbidity patterns with the risk of developing ADL and IADL stratified by sex.**

| **Multimorbidity patterns** | **Females n = 1,332** | **Males n = 785** |
| --- | --- | --- |
| **ADL** |  |  |
| Unspecific | Ref | Ref |
| Psychiatric | 1.42 (0.81, 2.51) | 1.41 (0.44, 4.46) |
| Cardio/Anemia/Dementia | 3.42 (1.42, 8.22) ** | 0.40 (0.05, 3.16) |
| Metabolic/Sleep | 1.35 (0.81, 2.24) | 1.52 (0.81, 2.85) |
| Sensory/Cancer | 1.34 (0.80, 2.25) | 2.42 (1.14, 5.15) * |
| MSK/Resp/GI | 1.38 (0.97, 1.97) | 1.09 (0.45, 2.62) |
| **IADL** |  |  |
| Unspecific | Ref | Ref |
| Psychiatric | 2.20 (1.02, 4.73) * | 1.13 (0.32, 3.93) |
| Cardio/Anemia/Dementia | 5.14 (2.02, 12.08) *** | 1.24 (0.41, 3.76) |
| Metabolic/Sleep | 1.84 (1.00, 3.37) | 1.19 (0.60, 2.35) |
| Sensory/Cancer | 3.45 (1.98, 5.99) *** | 0.75 (0.33, 1.67) |
| MSK/Resp/GI | 2.37 (1.52, 3.70) *** | 1.29 (0.55, 3.05) |

P-values: * < 0.05, ** ≤ 0.01, *** ≤ 0.001

Models adjusted by age, education level, marital status

Abbreviations: RRR – Relative Rate Ratio, GI – gastro-intestinal diseases, Resp – respiratory diseases, MSK – musculoskeletal, Cardio – cardiovascular diseases, CI – confidence interval

**Table S3. Association (RRR and 95%CI) of multimorbidity patterns with the risk of death and dropout from the study.**

| **Multimorbidity patterns** | **Total sample** | **Females** | **Males** |
| --- | --- | --- | --- |
| **Dead (ADL outcome)** |  |  |  |
| **Unspecific** | Ref | Ref | Ref |
| **Psychiatric** | 1.18 (0.58, 2.38) | 0.62 (0.21,1.83) | 2.43 (0.89,6.61) |
| **Cardio/Anemia/Dementia** | 4.48 (2.46, 8.15) *** | 4.96 (2.01,12.24) ^***^ | 4.55 (1.96,10.57) ^***^ |
| **Metabolic/Sleep** | 2.13 (1.43, 3.19) *** | 2.00 (1.11,3.60) ^*^ | 2.50 (1.42,4.41) ^**^ |
| **Sensory/Cancer** | 1.51 (0.94, 2.42) | 1.39 (0.76,2.52) | 1.61 (0.74,3.50) |
| **MSK/Resp/GI** | 1.49 (0.99, 2.23) | 0.94 (0.56,1.58) | 3.52 (1.82,6.84) ^***^ |
| **Dead (IADL outcome)** |  |  |  |
| **Unspecific** | Ref | Ref | Ref |
| **Psychiatric** | 1.05 (0.49, 2.22) | 0.51 (0.15,1.71) | 2.27 (0.79,6.53) |
| **Cardio/Anemia/Dementia** | 4.14 (2.21, 7.78) *** | 4.71 (1.92,11.52) ^***^ | 3.80 (1.50,9.61) ^**^ |
| **Metabolic/Sleep** | 2.06 (1.37, 3.10) *** | 1.63 (0.88,3.02) | 2.70 (1.51,4.81) ^***^ |
| **Sensory/Cancer** | 1.48 (0.89, 2.47) | 1.57 (0.80,3.05) | 1.38 (0.62,3.11) |
| **MSK/Resp/GI** | 1.50 (0.98, 2.30) | 0.99 (0.58,1.69) | 3.29 (1.64,6.60) ^***^ |
| **Dropout (ADL outcome)** |  |  |  |
| **Unspecific** | Ref | Ref | Ref |
| **Psychiatric** | 1.62 (0.93, 2.83) | 1.52 (0.77,2.98) | 1.82 (0.67,4.90) |
| **Cardio/Anemia/Dementia** | 2.35 (1.21, 4.55) * | 2.48 (0.93,6.59) | 2.57 (1.02,6.48) ^*^ |
| **Metabolic/Sleep** | 1.46 (0.97, 2.18) | 1.50 (0.84,2.65) | 1.40 (0.78,2.51) |
| **Sensory/Cancer** | 0.84 (0.49, 1.44) | 0.62 (0.30,1.25) | 1.36 (0.59,3.14) |
| **MSK/Resp/GI** | 1.12 (0.76, 1.64) | 1.06 (0.68,1.65) | 1.06 (0.47,2.41) |
| **Dropout (IADL outcome)** |  |  |  |
| **Unspecific** | Ref |  |  |
| **Psychiatric** | 1.62 (0.94, 2.79) | 1.58 (0.82,3.04) | 1.76 (0.65,4.73) |
| **Cardio/Anemia/Dementia** | 2.54 (1.19, 4.91) ** | 2.64 (1.04,6.71) ^*^ | 2.65 (1.04,6.80) ^*^ |
| **Metabolic/Sleep** | 1.34 (0.89, 2.02) | 1.35 (0.77,2.38) | 1.31 (0.73,2.37) |
| **Sensory/Cancer** | 0.91 (0.53, 1.57) | 0.82 (0.40,1.71) | 1.11 (0.48,2.57) |
| **MSK/Resp/GI** | 1.19 (0.81, 1.75) | 1.20 (0.77,1.85) | 1.07 (0.47,2.42) |

P-values: * < 0.05, ** ≤ 0.01, *** ≤ 0.001

Models adjusted by age, sex, education level, marital status

Abbreviations: RRR – Relative Rate Ratio, GI – gastro-intestinal diseases, Resp – respiratory diseases, MSK – musculoskeletal, Cardio – cardiovascular disease
